# EXAMINING AWARENESS AND USE OF COMPREHENSIVE GERIATRIC ASSESSMENTS IN CANCER CARE: A QUALITATIVE STUDY

**DOI:** 10.1093/geroni/igad104.0653

**Published:** 2023-12-21

**Authors:** Aaron Seaman, Karen Wickersham, Julia Rowland, Ciaran Fairman, James Hebert, Cynthia Thomson, Rosi Vogel, Daniela Friedman

**Affiliations:** University of Iowa, Iowa City, Iowa, United States; University of South Carolina, Columbia, South Carolina, United States; Smith Center for Healing and the Arts, Washington, District of Columbia, United States; University of South Carolina, Columbia, South Carolina, United States; University of South Carolina, Columbia, South Carolina, United States; University of Arizona, Tucson, Arizona, United States; University of Arizona, Tucson, Arizona, United States; University of South Carolina, Columbia, South Carolina, United States

## Abstract

Comprehensive geriatric assessments (CGAs) are critical tools for identifying the needs of older cancer survivors and planning effective treatment and follow-up care. Research on CGA implementation in clinical settings remains limited. We interviewed primary and cancer care providers to better understand current CGA practices, their potential, and the challenges of clinical use. Eight semi-structured interviews, lasting ~60 minutes, were conducted with participants identified through Cancer Prevention and Control Research Network partner listservs. Analysis was conducted iteratively. Providers reported that CGAs were useful and administering them with all incoming patients would be ideal but was not always possible. Instead, they used brief screening tools, which meant CGA domains were inconsistently assessed. Implementation was facilitated by clinic champions, multidisciplinary care teams, and institutional resources and buy-in. Findings from this research will provide potential solutions to addressing barriers to using CGAs related to staff capacity and competing demands, provider experience, and institutional priorities.

